# Reply: Gallstones, cholecystectomy, and the risk for developing pancreatic cancer

**DOI:** 10.1038/sj.bjc.6600693

**Published:** 2003-01-28

**Authors:** C Bosetti, E Negri, S Franceschi, C La Vecchia

**Affiliations:** 1 Istituto di Ricerche Farmacologiche ‘Mario Negri’, Via Eritrea 62, 20157 Milan, Italy; 2 Field and Intervention Studies Unit, International Agency for Research on Cancer, 150 Cours Albert-Thomas, F-69372 Lyon Cédex 08, France; 3 Istituto di Statistica Medica e Biometria, Università degli Studi di Milano, Via Venezian 1, 20133 Milan, Italy

Sir

In a combined analysis of the Nurses' Health Study and the Health Professional Follow-up Study, on a total of 206 women and 143 men with cancer of the pancreas, Schernhammer *et al*, (2002) did not find an increased risk of pancreatic cancer in relation to history of gallstones or cholecystectomy after adjusting for potential confounding factors. The issue of a possible association between gallstones or cholecystectomy and cancer of the pancreas is, however, still open to discussion, because several investigations reported an excess pancreatic risk in patients with gallstones. Apart from the papers quoted in Schernhammer *et al*, (2002), some excess risks were found in cohort studies from the United States (Bansal and Sonnenberg, 1996), Denmark (Johansen *et al*, 1996), and Sweden (Ye *et al*, 2001), and case–control studies from the United Kingdom (Cuzick and Babiker, 1989), Greece (Kalapothaki *et al*, 1993), and Israel (Schattner *et al*, 1997). The strength of the association, however, was variable across studies, and different potential confounding factors were not always taken into account.

In order to provide further information on the issue, we updated the analysis of a case–control study conducted in Italy between 1983 and 1992 (La Vecchia *et al*, 1990). Briefly, the study included 362 patients from the major teaching and general hospitals in Greater Milan with incident, histologically confirmed pancreatic cancer (229 men, 133 women, median age 59 years), and 1552 controls (1141 men, 411 women, median age 55 years) admitted to the same network of hospitals for acute, non-neoplastic conditions, unrelated to alcohol or tobacco consumption (33% traumas, 17% nontraumatic orthopedic conditions, 36% acute surgical diseases, and 14% other miscellaneous disorders). Less than 3% of cases and controls approached refused the interview.

Trained interviewers identified and questioned cases and controls using a structured questionnaire, including information on education and other socioeconomic factors, anthropometric measures, general lifestyle habits, such as tobacco and alcohol consumption, and a few selected indicator foods. The patients were also asked if they had a diagnosis of selected medical conditions, and the age at first diagnosis was recorded.

Odds ratios (OR) and corresponding 95% confidence intervals (CI) were estimated using unconditional multiple logistic regression models, including terms for age, education, tobacco consumption, body mass index, and history of diabetes.

Table 1

**Table 1:** Relation between pancreatic cancer and history of cholelithiasis among 362 cases and 1552 controls (Milan, Italy, 1983–1992)

	**Cases**	**Controls**	**OR^a^ (95% CI)**
History of cholelithiasis			
No	330	1430	1^b^
Yes	32	122	1.03 (0.67–1.59)
			
Time since diagnosis^c^ (years)			
<10	18	54	1.33 (0.74–2.40)
≥10	14	67	0.80 (0.43–1.50)

a Estimates from unconditional logistic regression models, including terms for age, sex, education, tobacco consumption, body mass index, and history of diabetes.

b Reference category.

c The sum does not add up to the total because of a missing value.

gives the distribution of pancreatic cancer cases and controls, and the corresponding ORs, according to history of cholelithiasis. Subjects with a history of cholelithiasis showed no increased risk of cancer of the pancreas (OR=1.03, 95% CI=0.67–1.59). The OR was 1.33 (95% CI=0.74–2.40) for subjects with a diagnosis of cholelithiasis less than 10 years before interview, and 0.80 (95% CI=0.43–1.50) for diagnosis 10 or more years before.

Thus, our findings are consistent with those of the Nurses' Health Study and the Health Professional Follow-up Studies (Schernhammer *et al*, 2002), and indicate that cholelithiasis is not materially associated with pancreatic cancer risk after major identified confounding factors have been considered. A modestly increased risk was observed 10 years after a diagnosis of cholelithiasis, but no greater excess risk can be found 10 or more years after. Thus, if any association exists, it is unlikely to be causal. The apparent association reported from several case–control studies can at least in part be because of a more accurate recall of gallbladder disease by pancreatic cancer patients. In our study, however, information on medical history proved satisfactorily reproducible (Bosetti *et al*, 2001), indicating that recall bias is unlikely to have played a major role. Other potential biases of this study should be limited, given the almost complete response rate, the administration of a standard questionnaire under similar conditions, and the same catchment area for cases and controls.
